# SrCe_0.9_Sm_0.1_O_3-α_ Compounded with NaCl-KCl as a Composite Electrolyte for Intermediate Temperature Fuel Cell

**DOI:** 10.3390/ma11091583

**Published:** 2018-09-01

**Authors:** Ruijuan Shi, Wei Chen, Wenli Hu, Junlong Liu, Hongtao Wang

**Affiliations:** 1 School of Chemical and Material Engineering, Fuyang Normal College, Fuyang 236037, China; rjshi@fync.edu.cn; 2 Department of science and health, Fuyang Preschool Education College, Fuyang 236015, China; chenwei20062002@163.com (W.C.); huwenli20062002@163.com (W.H.)

**Keywords:** defects, composite, electrolytes, hydrogen, fuel cell, conductivity

## Abstract

SrCeO_3_ and SrCe_0.9_Sm_0.1_O_3-α_ were synthesized using a high-temperature solid-state reaction method using Sm_2_O_3_, SrCO_3_, CeO_2_ as precursors, then the SrCe_0.9_Sm_0.1_O_3-α_-NaCl-KCl composite electrolyte was fabricated by compounding SrCe_0.9_Sm_0.1_O_3-α_ with NaCl-KCl and sintering it at a lower temperature (750 °C) than that of a single SrCeO_3_ material (1540 °C). The phase and microstructure of the samples were characterized by X-ray diffraction (XRD) and scanning electron microscopy (SEM). The conductivities of the samples were measured in dry nitrogen atmosphere using electrochemical analyzer. The conductivities of the SrCeO_3_, SrCe_0.9_Sm_0.1_O_3-α_ and SrCe_0.9_Sm_0.1_O_3-α_-NaCl-KCl at 700 °C were 2.09 × 10^−5^ S·cm^−1^, 1.82 × 10^−3^ S·cm^−1^ and 1.43 × 10^−1^ S·cm^−1^ respectively. The conductivities of SrCe_0.9_Sm_0.1_O_3-α_-NaCl-KCl composite electrolyte are four orders of magnitude higher than those of SrCeO_3_ and two orders of magnitude higher than those of SrCe_0.9_Sm_0.1_O_3-α_. The result of log*σ* ~ log*p*O_2_ plot indicates that SrCe_0.9_Sm_0.1_O_3-α_-NaCl-KCl is almost a pure ionic conductor. The electrolyte resistance and the polarization resistance of the H_2_/O_2_ fuel cell based on SrCe_0.9_Sm_0.1_O_3-α_-NaCl-KCl composite electrolyte under open-circuit condition were 1.0 Ω·cm^2^ and 0.2 Ω·cm^2^ respectively. Further, the obtained maximum power density at 700 °C was 182 mW·cm^−2^.

## 1. Introduction

Fuel cells have become one of the hot spots in the fields of new energy and environment protection. This is mainly because they have many advantages like high energy conversion efficiency, quiet operation, low cost and low pollution emission [1,2,3,4,5,6,7,8,9,10,11]. Cathode, anode and solid electrolyte are the vital components of the solid oxide fuel cell (SOFC). The key factor to design a high performance SOFC is the electrolyte. At high temperature, the perovskite-type solid electrolytes based on SrCeO_3_ exhibits predominant proton conduction under a hydrogen-containing atmosphere. They also have various applications in hydrogen sensor, fuel cell and membrane reactors, etc. Furthermore, partial substitution of the Ce^4+^ by a trivalent rare earth element, such as Eu^3+^, Y^3+^, Yb^3+^ and Lu^3+^, plays an important role in the improvement of the protonic conductivity of SrCeO_3_ when compared to pure SrCeO_3_ which exhibits low electronic conductivity [12,13,14,15,16,17,18]. Zhang et al. reported Yb doped SrCeO_3_ powders that were prepared using a low temperature gel combustion method and later studied the effect of ratio of citric acid to metals cations, oxidizer and calcination temperature on the properties of the SrCe_0.9_Yb_0.1_O_3-ö_ powders [14]. To investigate the effect of temperature on the total conductivity and hydrogen fluxes for europium-doped strontium cerate, Shrivastava et al. presented a numerical method to the defect chemical equations to model defect population in SrCe_0.95_Eu_0.05_O_3-α_ under hydrogen atmosphere [19]. However, research reveals that there are still challenges because the conductivity of trivalent cations-doped SrCeO_3_ is not high enough to meet the commercial application of solid oxide fuel cells.

In recent years, composite electrolytes based on doped ceria and alkaline carbonates or halides have been fabricated and exhibited enhanced ionic conductivities, as well as considerable cell power outputs compared to doped ceria [20,21,22,23,24,25,26]. Fu et al. suggested that the enhancement of ionic conductivity of gadolinium-doped ceria (GDC)-LiCl-SrCl_2_ composite electrolyte may be related to the interfaces between GDC and LiCl-SrCl_2_, which greatly facilitates the increase in the oxygen transfer routes [24,25].

Doped ceria electrolytes are conventional oxide ion conductors. However, there are only a few reports by Huang’s group and our group based on composite electrolytes of perovskite-type protonic conductors, such as BaCeO_3_ and SrCeO_3_ using inorganic salt for intermediate temperature fuel cell [27,28,29,30]. In addition, perovskite-type solid oxide-inorganic salts composite electrolytes have not been developed and investigated thoroughly.

In this work, SrCeO_3_ and SrCe_0.9_Sm_0.1_O_3-α_ were synthesized by a high-temperature solid-state reaction method. The SrCe_0.9_Sm_0.1_O_3-α_-NaCl-KCl composite electrolyte was fabricated by compounding SrCe_0.9_Sm_0.1_O_3-α_ with NaCl-KCl and sintering at lower temperature (750 °C). The phase and microstructure of the samples were characterized by X-ray diffraction (XRD) and scanning electron microscopy (SEM). The H_2_/O_2_ fuel cells were also fabricated and conductivities of the samples were measured using electrochemical analyzer in dry nitrogen atmosphere in an operating temperature range of 500–700 °C. The differences between previous studies are compared and addressed.

## 2. Experimental Section

SrCeO_3_(SC) and SrCe_0.9_Sm_0.1_O_3-α_(SCS) were firstly synthesized using solid-state reaction method. Stoichiometric amounts of raw materials, i.e., Sm_2_O_3_, SrCO_3_ and CeO_2_ were mixed together and ball milled thoroughly in the presence of anhydrous ethanol. The powdered mixtures were then calcined at 1300 °C for 5 h in air. After they cooled down to room temperature, the resulting powdered samples were finely ground and pelleted. Finally, the pellets were sintered at 1540 °C for 5 h to obtain SC and SCS.

Sodium chloride and potassium chloride were mixed together at a molar ratio of 1:1. Then they were ground thoroughly and placed in a ceramic crucible. After they were heat-treated two times at 700 °C, NaCl-KCl binary molten salt was achieved, which was then mixed with SrCe_0.9_Sm_0.1_O_3-α_ powders. The amount of NaCl-KCl molten salts was 20 wt % in the powdered mixture. The mixture was finely grounded and pelleted in a disk which was 18 mm in diameter and 2–3 mm in thickness. The pellet was subsequently calcined at 750 °C for 2 h to obtain SrCe_0.9_Sm_0.1_O_3-α_-NaCl-KCl composite electrolyte which were labeled as SCS-NK.

X-ray diffractometry (XRD, X’pert Pro MPD, Holland’s company, Amsterdam, the Netherlands) with Cu-kα radiation was carried out to do the structure and phase analysis of SC, SCS and SCS-NK samples and scanning electron microscopy (SEM, S-4700, Hitachi, Tokyo, Japan) was performed to obtain the surface and cross-sectional morphologies of the three pellet samples. The chemical physics changes and phase formation temperature of the SrCe_0.9_Sm_0.1_O_3-α_(SCS), SrCe_0.9_Sm_0.1_O_3-α_-NaCl-KCl (SCS-NK) and NaCl-KCl powder in the temperature range between room temperature to 800 °C at a heating rate of 15 °C·min^−1^ under nitrogen atmosphere were determined by thermogravimetry analysis and differential scanning calorimetry (TGA-DSC) (TGA-DSC, Universal V 3.7A, TA Instruments, New Castle, DE, USA), respectively.

The electrical conductivity of SC, SCS and SCS-NK pellets (thickness = 1.0–1.1 mm) were measured by means of AC impedance spectroscopy in a three-electrode system over the frequency range from 0.1 Hz to 1 MHz at 500–700 °C in dry nitrogen atmosphere using an electrochemical analyzer (CHI660E, Chen Hua company, Shanghai, China), the AC amplitude was 20 mV. The conductivity can be calculated from: σ = $\frac{L}{R\cdot S}$, where σ is conductivity, *L* is thickness, *R* is resistance and *S* is surface area of electrolyte pellet. The effect of different operating atmospheres and pressures on the electrical conductivity of SCS-NK was also examined. Oxygen’s partial pressure was controlled using mixtures of O_2_, N_2_ and H_2_, the partial pressure was also monitored in situ by an oxygen sensor placed in the experimental chamber(Melexis company, Brussels, Belgium) [26,27,28,29,30]. An oxygen concentration cell with platinum electrodes was developed to measure the electromotive force of SCS electrolyte membrane from 500 to 700 °C. A single cell using SCS-NK as an electrolyte membrane was performed in the presence of pure hydrogen acting as the fuel and oxygen as the oxidant. The fuel cell was tested using the linear scanning of current and voltage method within the CHI660E electrochemical analyzer [31,32,33,34]. Silver paste (areas = 0.5 cm^2^) covered on each side of the electrolyte membrane, they acted as the current collector and porous platinum was used as electrodes. The power density and open circuit voltage were recorded at 700 °C.

## 3. Results and Discussion

**Figure 1:**
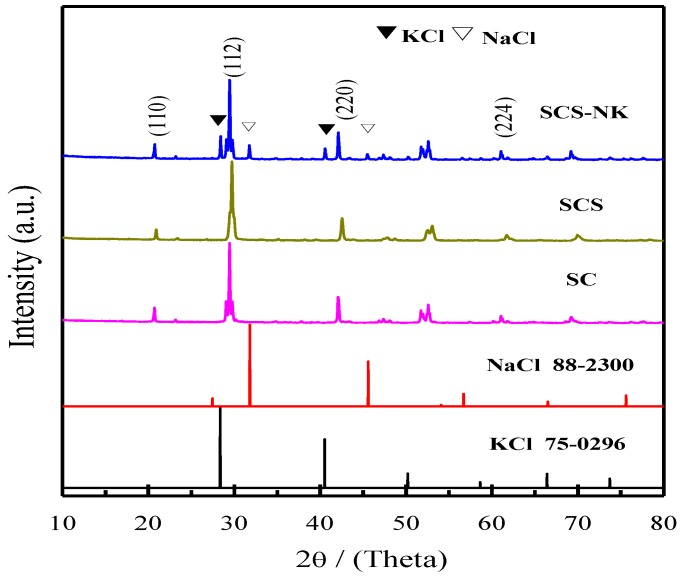
XRD patterns of SrCeO_3_(SC), SrCe_0.9_Sm_0.1_O_3-α_(SCS) and SrCe_0.9_Sm_0.1_O_3-α_-NaCl-KCl (SCS-NK) powder.

The powder XRD patterns of SrCeO_3_ (SC), SrCe_0.9_Sm_0.1_O_3-α_ (SCS) and SrCe_0.9_Sm_0.1_O_3-α_-NaCl-KCl (SCS-NK) are presented in Figure 1. It can be seen that all of three samples having the same main diffraction peaks at 20.75° (110), 29.48° (112), 42.18° (220) and 61.13° (224), this result is consistent with the standard data (JCPDS 082-2370) for the orthorhombic perovskite phase of SrCeO_3_. It was noted that some additional peaks related to NaCl and KCl salt phase appear in the SCS-NK composite, which reveals that NaCl-KCl salt in the form of crystalline was coexisting with SrCe_0.9_Sm_0.1_O_3-α_ and no reaction occurred between SrCe_0.9_Sm_0.1_O_3-α_ and NaCl-KCl salt during the process of heat treatment [30].

**Figure 2:**
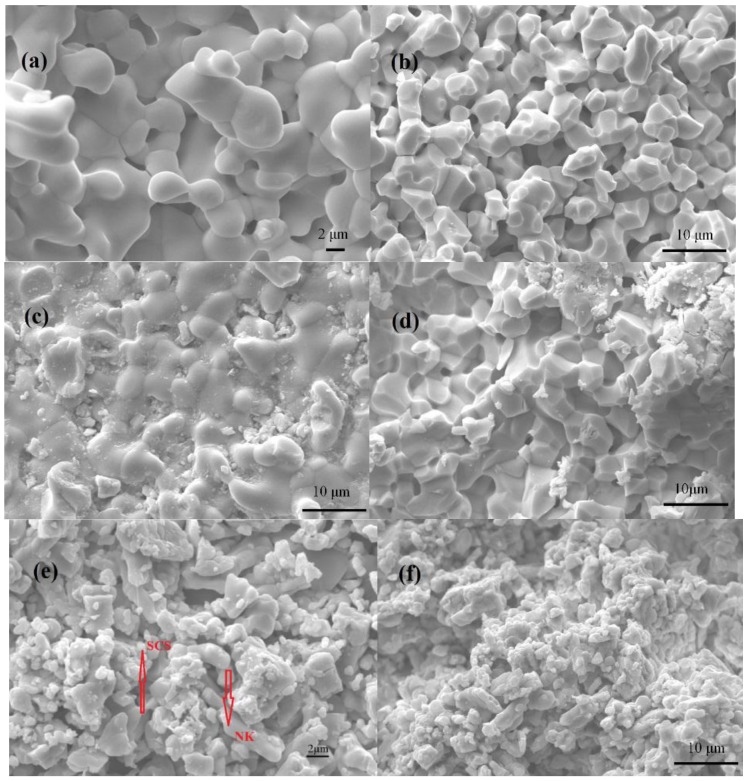
Surface (**a**,**c**,**e**) and cross-sectional (**b**,**d**,**f**) SEM images of SrCeO_3_(SC), SrCe_0.9_Sm_0.1_O_3-α_(SCS) and SrCe_0.9_Sm_0.1_O_3-α_-NaCl-KCl (SCS-NK) pellets.

Figure 2 shows surface (a,c,e) and cross-sectional (b,d,f) SEM images of SC, SCS and SCS-NK pellets. From Figure 2a,b, it can be observed that there are some closed pores on the surface and cross-sectional of SC, but this does not affect the electrical performance test. From Figure 2c,d, it can be seen that the SCS pellet shows more denser powder agglomeration than that of SC, However, a few closed pores still exists. From Figure 2e,f, it is obvious that the low-melting NaCl-KCl salt are closely connected to the surface of SCS powder and the SCS-NK composite electrolyte pellet was fully densified after calcining at 750 °C for 2 h, also there were no pores present [28,29]. This result is like the observation of the ceria-carbonates composite electrolytes in the study by Shawuti [20]. The SCS and NK have been indicated by arrows in Figure 2e. 

**Figure 3:**
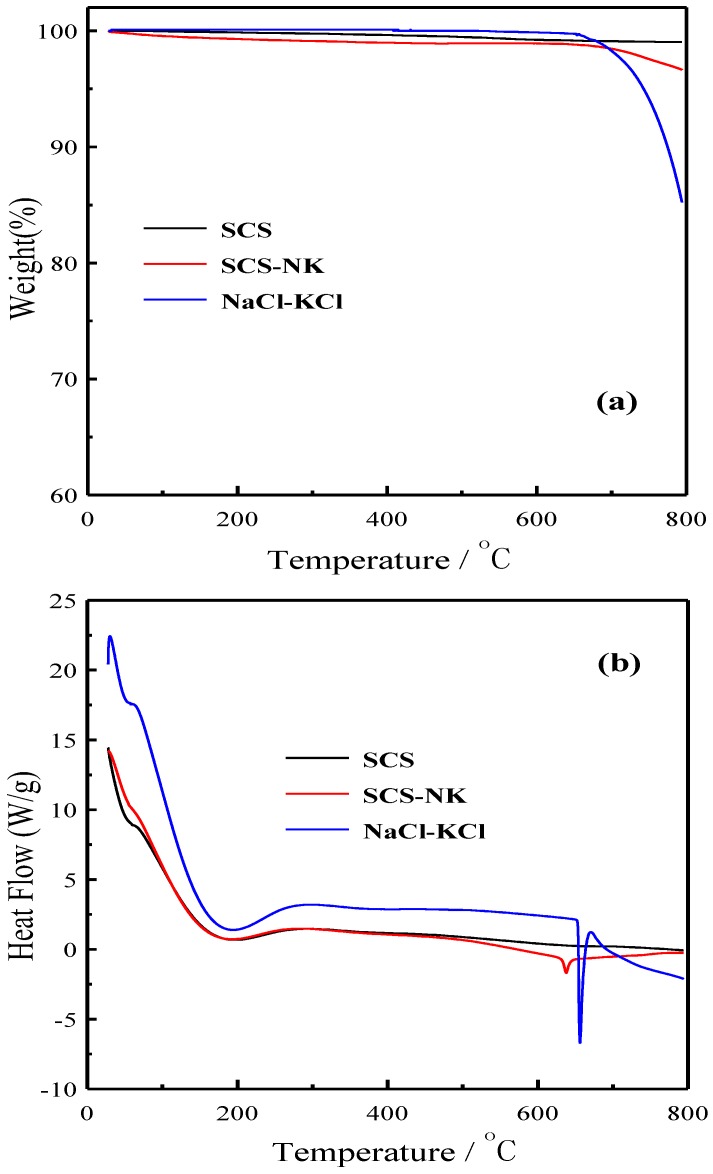
(**a**) TGA, (**b**) DSC curves of the SrCe_0.9_Sm_0.1_O_3-α_(SCS), SrCe_0.9_Sm_0.1_O_3-α_-NaCl-KCl (SCS-NK) and NaCl-KCl powder in nitrogen up to 800 °C.

Figure 3 shows the thermogravimetry analysis (TGA) and differential scanning calorimetry (DSC) curves of the SrCe_0.9_Sm_0.1_O_3-α_(SCS), SrCe_0.9_Sm_0.1_O_3-α_-NaCl-KCl (SCS-NK) and NaCl-KCl powder in the temperature range between room temperature to 800 °C at a heating rate of 15 °C·min^−1^ under nitrogen atmosphere, respectively. In Figure 3a, it is obvious that SCS has almost no weight loss in the whole test temperature range, while the TGA curves of the SCS-NK and NaCl-KCl show weight loss at ca. 720 °C and 670 °C, correspondingly. It can also be speculated that the stability of SCS-NK composite will decrease with the rise in temperature. The DSC curve of NaCl-KCl system has a vertical downward, strong endothermic peak at 655 °C from Figure 3b which is attributed to the melting process of inorganic salt. For the SCS-NK, the endothermic peak at 638 °C corresponds to the melting process changing from crystal to non-crystal in the composite [35,36].

**Figure 4:**
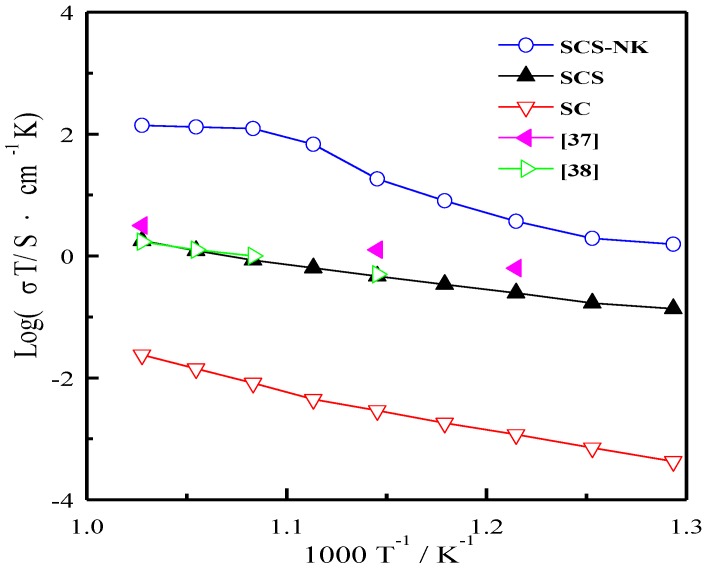
Temperature dependence of the conductivities of SrCeO_3_(SC), SrCe_0.9_Sm_0.1_O_3-α_(SCS) and SrCe_0.9_Sm_0.1_O_3-α_-NaCl-KCl (SCS-NK) in dry nitrogen atmosphere at 500–700 °C.

The variation in conductivities of SC, SCS and SCS-NK as a function of temperature in dry nitrogen atmosphere at 500–700 °C is depicted in Figure 4. It is evident that the conductivities were increased with the rise in temperature. SCS showed higher conductivity in comparison to SC and the highest conductivity was shown by SCS-NK composite electrolyte. The conductivities were 5.13 × 10^−7^ S·cm^−1^, 1.75 × 10^−4^ S·cm^−1^ and 1.39 × 10^−3^ S·cm^−1^ at 500 °C and 2.09 × 10^−5^ S·cm^−1^, 1.82 × 10^−3^ S·cm^−1^ and 1.43 × 10^−1^ S·cm^−1^ at 700 °C for SC, SCS and SCS-NK, respectively. The conductivities of SCS obtained in this work were compared with the literature data of some SrCeO_3_ based electrolytes sintered at high temperatures [37,38]. As shown in Figure 4, the conductivities of SCS were comparable to values of Sr_0.95_K_0.05_Ce_0.95_Y_0.05_O_3_ [37] and SrCe_0.9_Eu_0.1_O_3-α_ [38] under the same condition. It can be attributed to the high temperature solid state reaction method which could result in a dense electrolyte after calcination at 1300 °C and 1540 °C for 5 h, this can be confirmed by the SEM images of Figure 2c,d.

In our previous reports [26,30], SrCeO_3_-based electrolytes were mixed conductors of oxygen ion and electron hole under dry nitrogen atmosphere, whereas here they were mixed conductors of proton, oxygen ion and electron hole under wet nitrogen atmosphere. As we know, protons and oxide ion vacancies are the predominated defects under wet and dry conditions, respectively. In addition, the oxygen-ion diffusion in the conductors need high temperature, whereas protons are the smallest ions and the mobility of protons is much greater compared to that of other ions. Therefore, the conductivity of SrCeO_3_-based electrolytes is found to be higher in the wet nitrogen atmosphere when compared to dry nitrogen atmosphere.

It is clear that the ionic conductivities in SCS-NK composite electrolyte are four and two orders of magnitude higher than those of SC and SCS at 700 °C, respectively [13,15,16,28,29]. It can be concluded that the NaCl-KCl molten salt that was coated on the surface of SrCe_0.9_Sm_0.1_O_3-α_ and distributed along the grain boundaries, greatly increased the fast ion transfer routes and is beneficial for long distance transport of ions [23,29].

**Figure 5:**
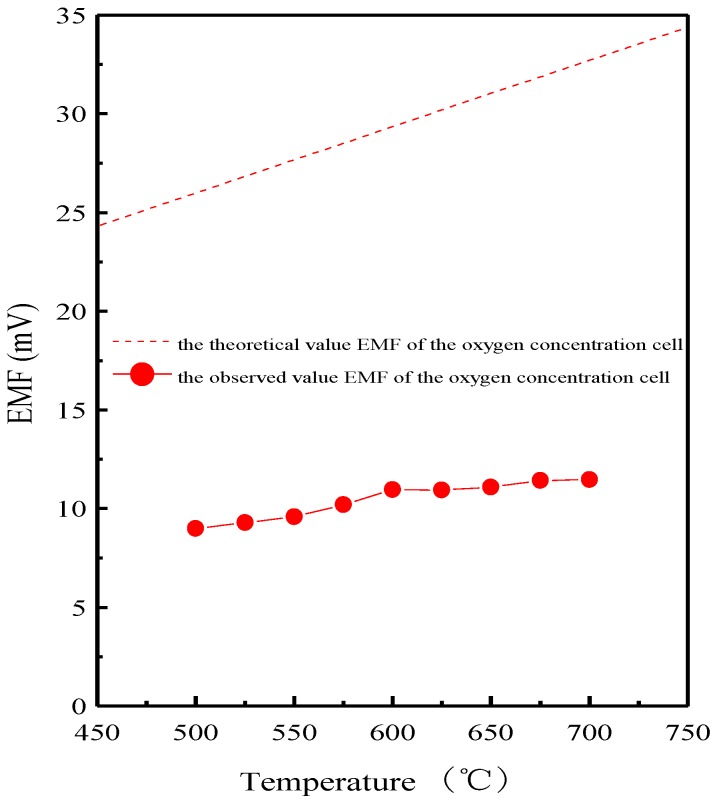
The electromotive force values of the oxygen concentration cell using SrCe_0.9_Sm_0.1_O_3-α_(SCS) as electrolyte in the temperature range from 500 to 700 °C.

An oxygen concentration cell using SCS as electrolyte membrane was investigated at operating temperatures between 500 and 700 °C. As shown in Figure 5, the observed electromotive force (EMF_obs_) values of the oxygen concentration cell were largely lower than the calculated electromotive force (EMF_cal_) values. The formula to calculate the electromotive force is the same as that described previously [30]. The results show that SCS electrolyte is not a pure ionic conductor, but a mixed conductor of oxide-ion and hole [12,39]. Transport numbers of oxygen ions in SCS electrolytes were 0.34–0.35 which were calculated from the EMF_obs_/EMF_cal_.

**Figure 6:**
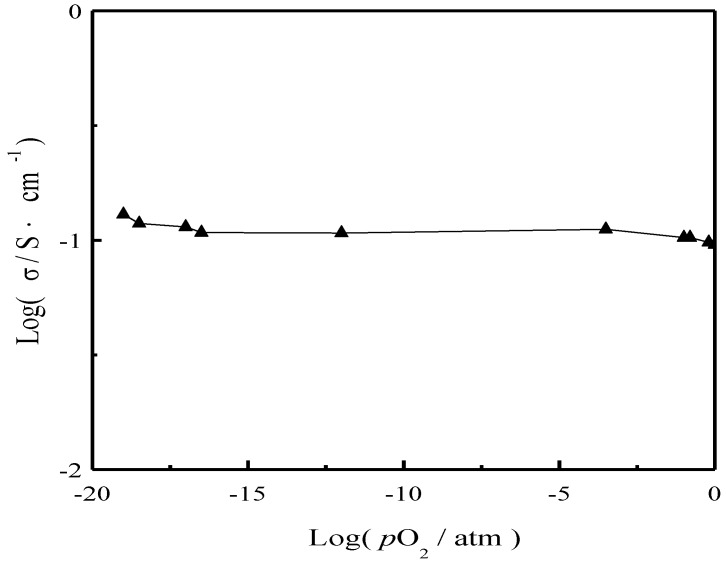
Plot of the conductivity of SrCe_0.9_Sm_0.1_O_3-α_-NaCl-KCl (SCS-NK) as a function of *p*O_2_ at 700 °C.

Figure 6 shows the conductivity of the SCS-NK composite measured at 700 °C as a function of oxygen partial pressure (*p*O_2_). As seen in Figure 6, the logσ values do not show any changes over oxygen’s partial pressure range of 10^−17^~10^−3^ atm, which suggests that SCS-NK composite electrolyte is virtually a pure ionic conductor. However, there were some minor deviations from linearity in conductivity in oxygen’s partial pressure ranging from 10^−1^^9^ to 10^−^^17^ atm and from 10^−^^3^ to 10^0^ atm, which reveals that SCS-NK composite electrolyte exhibits mixed conductivity and mainly ionic conducting properties over the oxygen partial pressure range [26].

**Figure 7:**
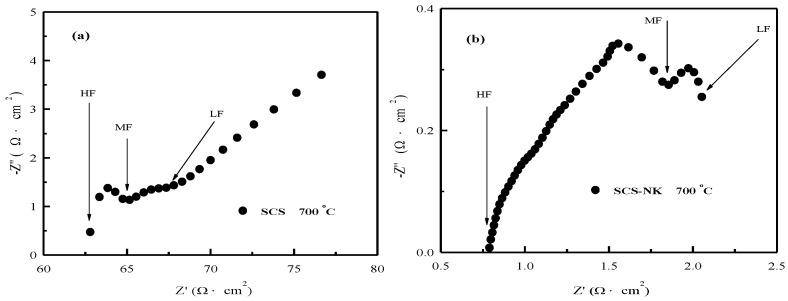
The impedance spectra of the single cells under open-circuit condition at 700 °C. (**a**) SrCe_0.9_Sm_0.1_O_3-α_(SCS) and (**b**) SrCe_0.9_Sm_0.1_O_3-α_-NaCl-KCl (SCS-NK).

The impedance spectra of SCS and SCS-NK under open-circuit condition at 700 °C are illustrated in Figure 7. The electrolyte resistance values were calculated by the difference in the semicircle diameter between high frequency (HF) and medium frequency (MF), and the polarization resistances values were obtained by the difference in the semicircle diameter between medium frequency (MF) and low frequency (LF). It can be seen that the electrolyte resistance and the polarization resistance for SCS were 3.2 Ω·cm^2^ and 1.8 Ω·cm^2^, respectively. The electrolyte resistance and the polarization resistance for SCS-NK composite electrolyte were 1.0 Ω·cm^2^ and 0.2 Ω·cm^2^, respectively [20,21,28,40]. The results indicated that NaCl-KCl molten salt in SCS-NK composite electrolyte, not only provides additional transport routes, but also increases the mobility of the carrier ions, which in turn leads to the decrease in activation energy, thus resulting in a higher electrical performance [40,41].

**Figure 8:**
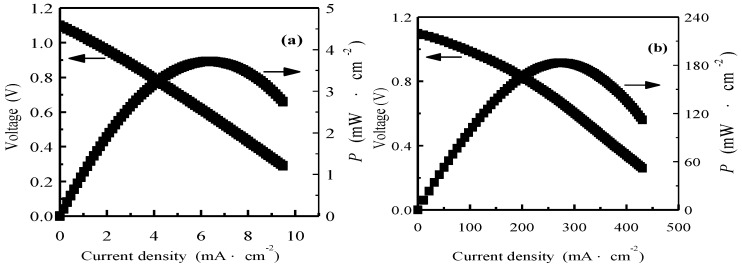
*I*-*V*-*P* curves of the single cell with SrCe_0.9_Sm_0.1_O_3-α_(SCS) (**a**) and SrCe_0.9_Sm_0.1_O_3-α_-NaCl-KCl (SCS-NK) (**b**) electrolyte at 700 °C.

The *I*-*V*-*P* curves of the single cells using SCS and SCS-NK as electrolyte membrane at 700 °C by using pure hydrogen as fuel and oxygen as oxidant is shown in Figure 8. The open circuit voltages of the single cells using SCS and SCS-NK as electrolyte were close to the theoretical values, which illustrated that the SCS electrolyte membrane was dense enough after sintered at 1540 °C for 5 h and was the same as the results of Figure 2c,d, on the other hand, it also revealed that SrCe_0.9_Sm_0.1_O_3-α_ is fully covered by and closely connected to NaCl-KCl salt to make the SCS-NK composite electrolyte pellet fully dense and facilitate charge carrier transportation. The maximum power density for the single cell using SCS-NK as electrolyte membrane was 182 mW·cm^−2^, which is more than 47 times higher than that of SCS (3.8 mW·cm^−2^). Fu et al. reported that the gadolinium-doped ceria (GDC)-LiCl-SrCl_2_ composite electrolyte shows a maximum power density as high as 260 mW·cm^−2^ at 550 °C which is much higher than that of GDC [24,25].In previous studies by our group, we have demonstrated that the hydrogen–oxygen fuel cells using SrCe_0.9_Gd_0.1_O_3-α_-NaCl-KCl and SrCe_0.85_Er_0.15_O_3-α_-NaCl-KCl composite as electrolytes generates the maximum power densities of 215 mW·cm^−2^ and 304 mW·cm^−2^ at 700 °C, respectively [28,29]. From the results of Figure 4 and Figure 8, we found that the ionic conductivity of SrCe_0.9_Sm_0.1_O_3-α_ was significantly improved when doped with Sm ions and the discharge performance of the SCS-NK composite electrolyte were superior to that of SrCe_0.9_Sm_0.1_O_3-α_ under the same test condition when further combined with NaCl-KCl salt. Such performance may be attributed to the higher ionic conduction of the composite electrolyte, which has been discussed above. However, the durability of the electrolytes have not been tested and will be done as future work.

## 4. Conclusions

In summary, the SrCe_0.9_Sm_0.1_O_3-α_-NaCl-KCl composite electrolyte was fabricated by compounding SrCe_0.9_Sm_0.1_O_3-α_ with NaCl-KCl and sintering at much lower temperature (750 °C) than that of single SrCeO_3_ materials (1540 °C). The conductivity of SrCe_0.9_Sm_0.1_O_3-α_-NaCl-KCl composite electrolyte was 1.43 × 10^−1^ S·cm^−1^ which is four orders of magnitude higher than that of SrCeO_3_ and two orders of magnitude higher than that of SrCe_0.9_Sm_0.1_O_3-α_ in dry nitrogen atmosphere at 700 °C. This can be because NaCl-KCl molten salt was distributed along the grain boundaries of SrCe_0.9_Sm_0.1_O_3-α_ which greatly increased the fast ion transfer routes and is beneficial for long distance transport of ions. The electrolyte resistance and the polarization resistance for SrCe_0.9_Sm_0.1_O_3-α_-NaCl-KCl composite electrolyte were 1.0 Ω·cm^2^ and 0.2 Ω·cm^2^ under open-circuit condition, respectively, and the obtained maximum power density was 182 mW·cm^−2^ at 700 °C, which is much higher than that of SrCe_0.9_Sm_0.1_O_3-α_ (3.8 mW·cm^−2^).
